# Operationalizing Heedful Interrelating: How Attending, Responding, and Feeling Comprise Coordinating and Predict Performance in Self-Managing Teams

**DOI:** 10.3389/fpsyg.2016.00362

**Published:** 2016-03-18

**Authors:** John Paul Stephens, Christopher J. Lyddy

**Affiliations:** Weatherhead School of Management, Organizational Behavior, Case Western Reserve UniversityCleveland, OH, USA

**Keywords:** coordination, heedful interrelating, self-managing teams, attention, responsiveness, tacit knowledge

## Abstract

Team coordination implies a system of individual behavioral contributions occurring within a network of interpersonal relationships to achieve a collective goal. Current research on coordination has emphasized its relational aspects, but has not adequately accounted for how team members also simultaneously manage individual behavioral contributions and represent the whole system of the team's work. In the current study, we develop theory and test how individuals manage all three aspects of coordinating through the three facets described in the theory of heedful interrelating. We operationalize the facet of contributing as distributing attention between self and others, subordinating as responsively communicating, and representing as feeling the system of the team's work as a cohesive whole. We then test the relationships among these facets and their influence on team performance in an experiment with 50 *ad hoc* triads of undergraduate student self-managing teams tasked with collectively composing a song in the lab. In analyzing thin-slices of video data of these teams' coordination, we found that teams with members displaying greater dispersion of attentional distribution and more responsive communicating experienced a stronger feeling of the team as a whole. Responsive communication also predicted team performance. Accounting for how the three aspects of coordinating are managed by individual team members provides a more critical understanding of heedful interrelating, and insight into emergent coordination processes.

## Introduction

The growing complexity and uncertainty of the modern economy has necessitated increasing reliance on team-based structures such as self-managing teams (SMTs; Lawler et al., 1995; Reynolds, 2006) that are more flexible and responsive to environmental changes. SMTs are groups of interdependent individuals that have the authority and power to determine how to interrelate their efforts to complete some task (Cohen et al., 1996). As such teams rise in popularity, it becomes increasingly important to understand how they function and what influences their effectiveness. Lacking traditional formal supports for coordination, such as external hierarchy or plans (Lawrence and Lorsch, 1967), SMT performance instead depends on effective and emergent coordination processes. One way of explaining how effective coordinating emerges within a team is heedful interrelating (Weick and Roberts, 1993), where individual team members are aware of how their efforts are purposefully contributing toward the team's goal (Dougherty and Takacs, 2004).

Heedful interrelating was initially conceptualized from observations of coordination processes in the high-risk context of an aircraft carrier flight deck, where small errors could lead to catastrophe (Weick and Roberts, 1993). However, scholars claim that heedful interrelating is important in other organizational contexts where “task interdependence is high and task programmability is low” (Bijlsma-Frankema et al., 2008, p. 25). These conditions are met in SMTs, whose members rely on each other to autonomously and fluidly respond to each other. While case studies suggest that heedful interrelating can influence team performance in a range of settings (e.g., Druskat and Pescosolido, 2002; Hargadon and Bechky, 2006; Kolbe et al., 2009), we know little about the psychological mechanisms that make interrelating heedful or heedless. This limits our overall understanding of how effective patterns of coordinating emerge among a team of self-managing individuals, and how these can be managed.

Heedful interrelating has three main facets (Weick and Roberts, 1993), but several theoretical, empirical, and practical roadblocks limit our understanding of them. The first facet is contributing, or the actions that participants provide for each other; the second is subordinating, or shaping those actions so that they fit with the actions other participants provide; and the third is representing, or envisioning the system of collective work being realized by the team as a whole. With continuous awareness of this co-created system of work, individuals can heedfully shape their contributing and subordinating. While this original theorizing has prompted empirical research on how they impact emergent coordination, there has been little theoretical advancement of the concept that would deepen our understanding of heedful interrelating and help to specify its generalizability across contexts. Additionally, empirical work has not used comparable operationalizations of the three facets, instead offering indistinct operationalization (e.g., Yoo and Kanawattanachai, 2001) or avoiding this altogether (e.g., Kolbe et al., 2009). Third, we know something about the contextual factors that can shape heedful interrelating (e.g., Druskat and Pescosolido, 2002; Hargadon and Bechky, 2006), but little about how team members establish and maintain heed while interrelating. This limits our understanding of how SMTs effectively manage their coordinating.

In this article, we address these issues by developing and testing operationalizations of the three facets of heedful interrelating that are based in concepts generalizable across interdependent team contexts. First, we outline how “heed” can be understood as putting into action executive attention, which helps individuals balance multiple pieces of information in a deliberate fashion (Ocasio, 2011). We then hypothesize that since individual SMT members do not have strong external mechanisms to guide their coordinating (e.g., plans, protocols, or schedules), contributing occurs via individuals distributing their attention across their own actions and the actions of others. By distributing their attention across self and other, individuals can access the information needed to be responsive, or to shape their behaviors in ways that fit others'. As individual team members distribute their attention, and responsively communicate with each other while coordinating, their concerted actions form a meaningful pattern of collective action that participants represent as a tacit sense or feeling of acting as unit. We provide evidence for this theorizing in the form of observational and questionnaire data from members of 50 *ad hoc* SMTs coordinating in a song-composition task. By examining our operationalizations in a controlled lab setting, we aimed to remove some of the “noise” found in field settings, and to more stringently test our theorizing in the context of the emergent coordinating of new, *ad hoc*, SMTs assembling for the first time.

Our study advances our understanding of how effective coordinating is heedfully practiced within SMTs in two ways. First, grounding our theorizing in attention offers an empirically tractable mechanism that is central for information-processing in individuals across situations (Desimone and Duncan, 1995; Woodman and Luck, 2003), and for behavior across organizational contexts (Ocasio, 1997, 2011). This provides a common thread for researchers and team members to better understand and practice coordinating across a range of interdependent work contexts. Second, by articulating and testing how individual-level attentional distribution is the basis for other concepts such as responsiveness and felt, tacit knowledge, we link heedful interrelating to a larger network of relational and individual-level constructs. Introducing such mechanisms enriches the initial conceptualizations of how individuals emergently develop cohesive team functioning in ways that motivate newer and more complex questions and insights for researchers.

## Theoretical background and hypotheses

SMTs are defined by an ability to plan and execute how they will coordinate. It is the members of the SMT, rather than external supervisors or managers, who monitor each other, get to decide what counts as appropriate behavior, and otherwise influence each other to coordinate work that meets both individual and collective goals (Stewart et al., 2012). From this perspective, SMTs are important to study because they increase the flexibility of the organizations in which they are embedded (Van der Vegt et al., 2010) and serve as a microcosm of the self-organizing collaborative work that drives innovations in today's economy (e.g., O'Mahony and Lakhani, 2011; Adler, 2015). What is central to this emergent self-organizing is coordination, or the “temporally unfolding and contextualized process of input regulation and interaction articulation to realize a collective performance” (Faraj and Xiao, 2006, p. 1157).

A review of the research on SMTs reveals that we know relatively little about the ongoing, emergent dynamics of how SMTs actually practice their coordinating. Historically, research has either focused on the influence of contextual features on SMT processes (e.g., Cohen, 1994; Cohen and Ledford, 1994), and outcomes (e.g., Cordery et al., 1991), or the presence or absence of coordination patterns, rather than the qualities of those patterns (e.g., Pearce and Ravlin, 1987; Cohen et al., 1996). In trying to explain the coordination among SMT members, distal variables such as turnover are considered (Van der Vegt et al., 2010), rather than individual team members and their actual patterns of action. Recent work on SMTs continues to focus on how emergent properties of the group (Stewart et al., 2012), or the distribution of individual-level inputs (e.g., personality traits; Humphrey et al., 2011) affect performance. Without understanding SMT coordination practices, it is unclear how features like team membership or context impact the interrelating of actions that ultimately translates into collective performance.

We suggest that a key approach to understanding the emergent dynamics of coordinating in SMTs is applying the lens of heedful interrelating, which simultaneously captures the three central aspects of coordinating found in Faraj and Xiao's (2006) definition. These aspects include “inputs,” their “interaction articulation,” and the “collective performance” that results. Along with other coordination scholars (e.g., Okhuysen and Bechky, 2009), we use this definition because it offers a comprehensive multilevel view of coordination that accommodates its processual and emergent nature. While heedful interrelating and its three facets have not been formally linked to the components in Faraj and Xiao's (2006) definition, connecting the two conceptualizations demonstrates the broad usefulness of heedful interrelating for coordination research. First, “inputs” can be described as the “parts” that comprise the work of the team. Coordination research has described “parts” as specialized roles and divisions of labor in interdependent work, and the specialized roles individuals hold in these divisions (Thompson, 1967; Heath and Staudenmayer, 2000; Bechky, 2006; Gittell and Douglass, 2012). However, since roles are negotiated as people coordinate (Bechky, 2006; Madden et al., 2012), what ultimately matters are the actions that individuals produce as they enact their roles, or the contributing that forms the basis of heedful interrelating.

Second, “interaction articulation” refers to the interrelating among the parts, which comprises the core of coordinating. People need to collaborate in order to transform diverse inputs into joint understandings (Dougherty, 1992; Hargadon and Bechky, 2006) and benefit from high-quality communication in these interactions (Gittell, 2002; Gittell et al., 2010). Similarly, subordinating describes how contributing is conducted in ways that “supplement, assist, and become defined in relation to the imagined requirements of joint action” (Weick and Roberts, 1993, p. 365). Third, the “collective performance” realized in coordinating is based in subordinating contributions to each other in ways that allow them to fit and intermesh. The representing described in heedful interrelating captures how individual team members can envision this collective performance while they coordinate. In one example from the aircraft carrier flight deck, a pilot trying to land can represent or treat the instructions from air tower monitor personnel, the men on the landing signal officer's platform, and the men monitoring the aircraft off the ship as if they from one entity, although their sources are relatively independent (Weick and Roberts, 1993).

While the concept of heedful interrelating captures the three main aspects of coordinating, much of the research on coordination has focused primarily on how individuals manage their roles and actions (contributing parts) and the relationships among those parts (subordinating). For example, Heath and Staudenmayer (2000) detail how individuals often focus on knowledge of the parts involved in coordination, to the detriment of appreciating the relationships among those parts. Bechky (2003, 2006) looks at how individuals in different roles mutually negotiate their specialized, role-based understandings. Gittell's (2001, 2002) work on relational coordination takes a close look at the quality of how individuals enact their roles in relation to each other (e.g., the timeliness of communication, its helpfulness in problem-solving, and the degree of shared knowledge and goals). Ultimately, all of these examples aim to understand how the parts and their relationships relate to a superordinate task or collective performance. However, when the “whole” is referenced, its integration with the parts and their relationships is often left unclear. For example, pursuing both individual-level and team-level goals are important for successful teamwork, but we understand more about the need to choose one over the other when they conflict, rather than how individuals manage to pursue both simultaneously (cf. DeShon et al., 2004).

Heedful interrelating is therefore a useful concept since it interconnects all three core aspects of coordinating while accounting for the emergent nature of coordinating in SMTs. Much of the research on teams has traditionally explained team performance in terms of inputs (e.g., knowledge, traits, and behaviors), mediating processes that transform those inputs (e.g., coordinating), and then resultant performance outcomes (Ilgen et al., 2005). Heedful interrelating instead suggests that we should focus on how inputs such as individual heedfulness are manifested in the ongoing practice of coordinating, rather than on the precursors to some process. Teams researchers often distinguish between transition and action phases of team performance, where the former refers to times set aside for planning and evaluation, and the latter refers to times where the team puts those plans into action to accomplish their goals (Marks et al., 2001). By contrast, heedful interrelating calls on us to pay more attention to how well SMT members' actions evidence the plans, knowledge, and other inputs that can potentially shape behavior, rather than the inputs themselves. Ultimately, the ways these inputs are enacted create the conditions for further coordinating. This suggests that the emergent, self-directed conditions found in SMTs, and assumed in heedful interrelating theory, are consistent with the IMOI (input-mediator-output-input) framework which draws our attention to potential causal feedback loops, where an output at one time point can later on serve as an input (Ilgen et al., 2005).

By taking a performative view of coordinating—one that addresses how individuals simultaneously manage the parts, their relationships and the whole system of work—heedful interrelating has been applied to research on collaborative design work for consumer products (Hargadon and Bechky, 2006), student project teams (Yoo and Kanawattanachai, 2001; Bijlsma-Frankema et al., 2008), and SMTs in contexts ranging from a dog food plant to a mining company (Druskat and Pescosolido, 2002). However, more research is warranted to address the poor distinction among the three facets of heedful interrelating in extant operationalizations. Some empirical examples demonstrate how individuals care for their interactions and relationships, but do not distinguish among contributing, subordinating, and representing (e.g., Druskat and Pescosolido, 2002). Other operational definitions distinguish between heedful contributing, subordinating and representing, but the actual data seem to conflate qualities of multiple facets, e.g., “contribution” sounds like “representation” when the former includes sharing of information about who was doing what and how their actions fit together (Crowston and Kammerer, 1998). In other examples, survey measures of “collective mind” (e.g., “team members carefully interrelated actions to each other;” Yoo and Kanawattanachai, 2001) or qualitative codes that measure heedful interrelating (e.g., “teaching others;” “verbalizing own behavior;” or “considering the future;” Kolbe et al., 2009) do not distinguish among contributing, subordinating, or representing. Understanding the interconnected yet distinct nature of these facets is an essential step toward understanding how heedful interrelation impacts team functioning. In order to better understand the emergent coordinating of SMTs through the lens of heedful interrelating, we develop theory and hypotheses that describe attention as the basis for heed, and suggest contributing, and subordinating facilitate representing.

### Operationalizing heed as attentional behavior

Understanding heedful interrelating requires defining “heed,” which refers to attending to how something is being done while doing it, e.g., when someone drives carefully, or a clown artfully trips over himself to draw a laugh (Ryle, 1949). Ryle described heed as when “thinking what I am doing, I am doing one thing and not two” (1949, p. 32), which closely reflects modern notions of executive attention. Attention refers to a set of interconnected cognitive processes for selecting and registering information (Posner and Petersen, 1990, p. 26), and executive attention in particular refers to “the ability to control attention to ongoing cognitive processes” (Bosco et al., 2015, p. 2). Simultaneously managing multiple pieces of information via executive attention facilitates behavioral regulation (Posner and Rothbart, 2009; Ocasio, 2011). Executive attention underpins behavioral adaptation to suit contextual conditions and facilitate goal-fulfillment (Bandura, 1991; Baumeister and Heatherton, 1996), which is an integral aspect of heed.

In contrast, heedlessness (alternatively described as mindlessness) may begin with uncontrolled attention that is distracted or diminished (Ashforth and Fried, 1988; Brown et al., 2007). This can manifest in automatic, poorly-regulated behavior that inhibits task and goal achievement (Baumeister and Heatherton, 1996). When individuals are less attentive to how their behaviors will suit task and situational demands they struggle to behave appropriately (e.g., Carver and Scheier, 1982; Smallwood and Schooler, 2015), and thus impede organizationally-relevant performance (Reason, 1990; Jett and George, 2003; Warm et al., 2008; Westbrook et al., 2010; Mrazek et al., 2011; Smallwood et al., 2011). Based on these understandings, we begin our hypothesis development by focusing on the attention demonstrated in the individual actions that comprise coordinating in SMTs.

#### Contributing as behaviorally distributing attention

We specify heedful contributing as being comprised of behaving in ways that demonstrate some balance in attention to the self and others. Information-processing models of behavioral regulation describe how directing attention to behavior and desired goals and standards enables determination of whether behaviors meet those standards (Manz and Sims, 1980; Carver and Scheier, 1982). If we view heed in terms of executive attention, then the actions that individuals contribute when heedfully interrelating should reflect the context in which they emerge (Ocasio, 2011), and an interdependent context demands that individuals are aware of what others are doing (Victor and Blackburn, 1987) and their contributions to collective objectives (Brett et al., 1999; Langfred, 2004).

In teams, a given individual coordinates with other people. In such a situation, each individual must carefully track multiple others' efforts, suggesting that heedfully contributing individuals must continually balance attention across the targets of self and others. By contrast, heedless contributing would describe behaving in ways mainly attentive to the self and neglectful of the other. We therefore characterize heed as relative, rather than absolute, attentional distribution to self and others. We assume that individuals must always take in information about their own actions to facilitate self-regulation, and are always exposed to others' contributions. Executive attention helps individuals to manage multiple goals and stimuli simultaneously when there is little predetermined structure (Parasuraman, 1998; Fernandez-Duque et al., 2000), so we theorize that heedful contributing involves individuals toggling their attention back and forth between self and others while coordinating. Without a strong *a priori* basis for predicting an optimal distribution, it seems reasonable to assume that heedful contributing involves equally distributing attention across self and others.

#### Subordinating as communicating responsively

Executive attention helps individuals to manage their actions in ways that are appropriate to their interdependent context. This shaping of individual actions so that they are appropriate or adaptive to the demands of the team context describes subordinating, which can be understood in terms of how responsive individuals are to each other. Responsiveness is defined as “the processes through which relationship partners attend to and respond supportively to each other's needs, wishes, concerns, and goals, thereby promoting each other's welfare” (Reis and Clark, 2013, p. 400). Responsiveness begins with some behavioral expression by one participant that must then be attended to by another participant (Davis and Holtgraves, 1984). Successful relationships involve participants doing more “turning toward” each other, rather than “turning away” (Gottman, 1998). However, since “one's own needs, goals, and wishes…influence motivation and perception” (Reis and Clark, 2013, p. 402), being responsive involves attention to both self and other. The responsiveness of a contribution is further defined by qualitative features such as the relevance to the preceding communication, and the degree of timeliness for others (Davis, 1982).

While timeliness is just one feature of responsiveness, it is an important one. Being responsive in the course of coordinating demands that team members engage in a range of behaviors, such as mutually monitoring each other's changing needs, proactively providing back-up behaviors or assistance and feedback (Marks et al., 2001; Rico et al., 2008; Rosen et al., 2011), and respectfully offering up solutions to problems (Gittell, 2001, 2002). However, for teams that must emergently shape their coordinating, while actions such as redistributing efforts are important, it is the speed and timeliness of such actions that predict effective team performance, more so than their frequency (e.g., Waller, 1999; Gittell, 2002). More recent research suggests that when team members have a common understanding of how they should manage the time used to perform their collective task, they perform better as a team (Mohammed and Nadkarni, 2014). Heedfully subordinating can thus be more precisely defined in terms of quickly contributing in ways that are appropriate, relevant, and helpful for others across a team.

#### Representing as feeling wholeness of teamwork

Heedful individuals need to attend to their own needs and behaviors, as well as those of their teammates, in order to have the necessary information for developing responsive behaviors. While contributing and subordinating describe certain qualities of how individuals manage their dyadic interactions, representing refers to how individuals can have the “group in their head and use it for continued guidance of their own individual action” (Weick, 1993, p. 640). Specifically, representing refers to how individuals treat their multiple interactions with diverse team members as if all those interactions were of the same kind or source. In treating the various contributions being provided by themselves and others as if they were of a cohesive group, individuals can begin to experience their collective performance as if it was of one piece, rather than as a set of disjointed contributions. In turn, by treating these diverse interactions as if they were from a cohesive group, individuals are more likely to actually co-create a cohesive, unified group performance.

Representing can thus be understood as a process of assuming a certain degree of cohesiveness among team members' diverse contributions, and shaping contributing and subordinating in terms of this assumed cohesiveness. This then becomes realized in collective performance, reinforcing a sense of cohesiveness. Experiencing group performance as if it was of a cohesive whole is thus “both the product and condition of actions of individuals” (Asch, 1952, p. 251). This can be seen in how members of the Mann Gulch wildland firefighting team experienced different outcomes in battling an out-of-control blaze, based on their different experiences of the work of the group (Weick, 1993). Although, the team as a whole encountered the fire, the leader “presumed [the group] still existed,” while several of his team members “took less notice of one another” (p. 638). In treating his group as if it was still intact, the leader was motivated to develop an escape route for the group and was able to survive, while team members who treated each other as if they were disconnected began to break off, fleeing into the fire.

We suggest that as individuals emergently coordinate their actions, their representing takes the form of feeling the form of the team's work as a more or less cohesive whole. Treating the work of multiple team members as if they cohere in an emergent collective performance involves recognizing the patterns made by these diverse contributions, which have their own holistic and tacitly known qualities (Parmigiani and Howard-Grenville, 2011). Tacit knowledge refers to what we implicitly know through our direct, bodily engagement with the world (e.g., the know-how of being able to ride a bicycle), as distinct from the explicit knowledge we can articulate in our speech and writing (e.g., instructions on how to assemble a bicycle; Polanyi, 1966). Understanding something as a whole involves integrating the myriad parts involved (e.g., the multi-source directives to take off and land that a pilot encounters) in ways that are non-conscious and often only described in terms of a “feeling” (e.g., Dewey, 1934; Tsoukas and Vladimirou, 2001; Johnson, 2008). For example, Weick and Roberts (1993) describe how pilots monitoring the catapult crew guiding their landing “keep asking themselves questions like, ‘Does *it* feel right?’ or ‘Is the rhythm wrong?”’ with the “it” referring to the “joint situation” (p. 363) or the collection of diverse signals being generated by the pilot himself, the people on the deck and those directly in front the plane.

Representing can thus be understood as a felt quality of the unity or cohesiveness of team-level performance that informs future, ongoing coordinating. In this way, we see feeling as describing how various concepts of team functioning and knowledge, such as group cohesion, systems monitoring, and situational awareness are actively experienced in the course of coordinating. Group cohesion refers to the extent to which individuals see group goals as their own (Stewart et al., 2012), and it positively predicts team performance (Gully et al., 1995; Beal et al., 2003). Though varied, some measures of group cohesion ask about the extent to which people feel they are really part of a group (Seashore, 1954), or if the team seems unified in its goal pursuit (Carron et al., 1985). Systems monitoring, on the other hand, refers to tracking how internal team resources, and external environmental conditions all relate to team goals (Marks et al., 2001). Similarly, situational awareness refers to having an integrated picture of all the various elements of the environment, and understanding what that picture means for current and future actions (Endsley, 1995). These latter constructs do not refer to feeling, and are defined in terms of consciously tracking multiple “parts.” However, since both constructs describe the synthesis of elements into patterns, we theorize that these patterns have tacit qualities that are known through feeling.

**Figure 1:**
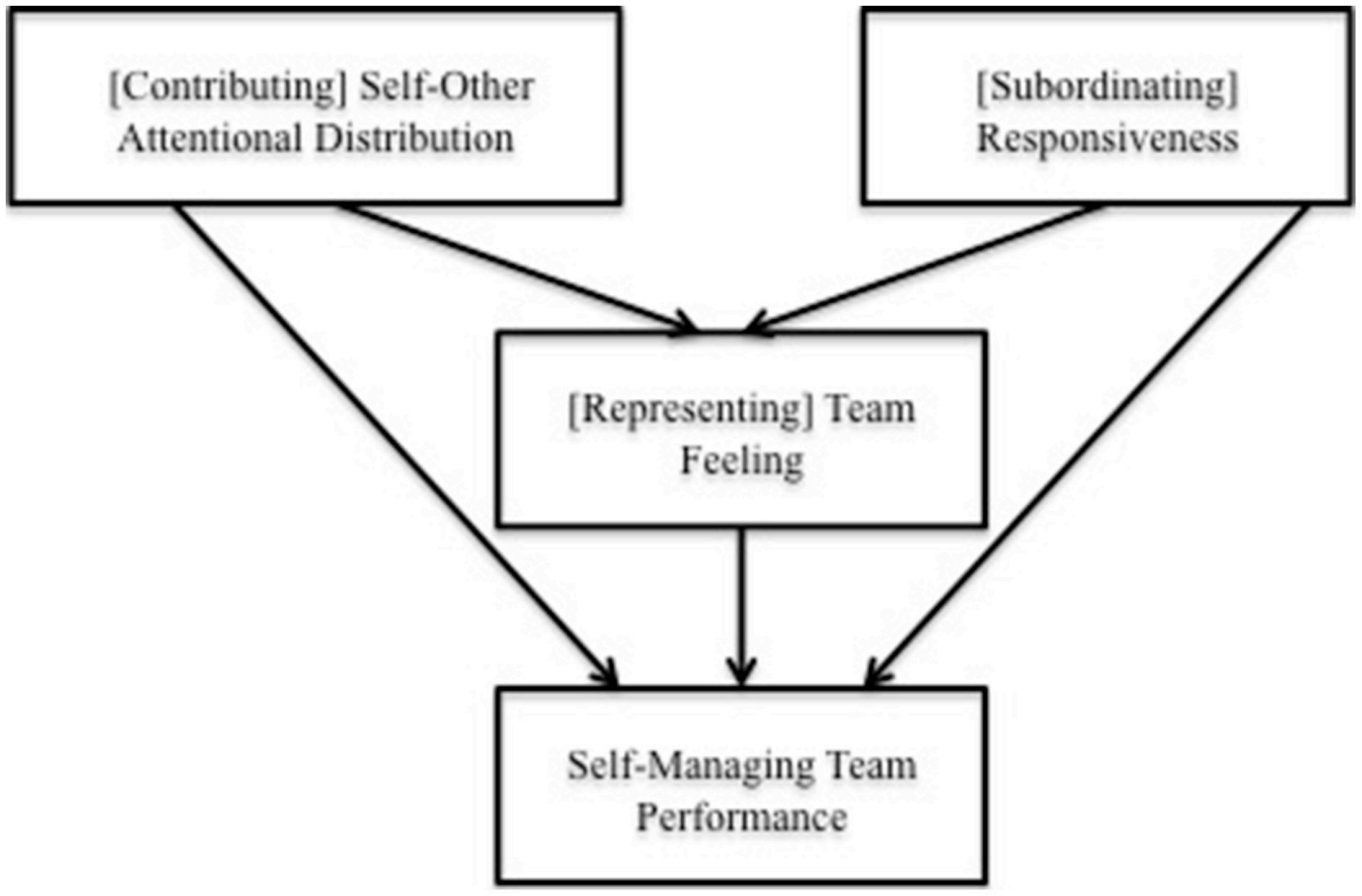
**Theorized model of relationships between operationalizations of contributing, subordinating, representing, and SMT performance**.

These enriched conceptualizations and operationalizations add specificity to the facets of heedful interrelating, and link them to constructs that can be found across a range of team settings. This theorizing also helps us develop hypotheses about the relationships among contributing, subordinating, and representing, to better explain how heedful interrelating emerges in SMTs. These hypotheses are summarized in Figure 1.

Much of the research on heedful interrelating assumes that all three facets must co-occur in order for a team to effectively coordinate (e.g., Bijlsma-Frankema et al., 2008; Kolbe et al., 2009). However, individuals beginning to coordinate as an SMT for the first time may not necessarily be able to heedfully contribute, subordinate, and represent right away (even though well-developed groups may also be unable to do so; Weick and Roberts, 1993). As members of an SMT begin to coordinate with a view to developing collective performance, they must first contribute actions based on some balance of attention to their own actions and the actions of others. With enough team members similarly drawing on attention to multiple, overlapping sets of actions, it should be possible for team members to begin to represent those actions as a coherent pattern. With enough team members demonstrating such attentional distribution, fellow team members may mimic such behavior, spreading it across the team, and ultimately yielding a team with a heedful attentional distribution (Fowler and Christakis, 2010). Alternatively, team members who display imbalanced attention may influence their colleagues and produce collective heedlessness. Excessively self-focused individuals may inspire others to withdraw their attention from their teammates, producing a team with a heedless attentional distribution, and a feeling that the team's work is a set of disjointed parts.

In terms of self-regulation, individuals who excessively focus on others without a complementary focus on the self may inordinately rely on others as an external standard by which to reference the self. Individuals tend to focus on whatever aspects of themselves are made salient by the immediate social environment (e.g., McGuire et al., 1979; Hinkley and Andersen, 1996). Thus, an excessive focus on others may induce judgments of one's behaviors in terms of how they stand relative to others. This may be accompanied by less simultaneous consideration of one's own needs, goals, and capabilities on their own terms, limiting a fuller appreciation of the interdependent, rather than dependent, nature of their team context. In observing excessively other-focused individuals, team members may mimic those individuals' dependent, reactive behaviors, and fail to provide meaningful contributions to each other as a group. The preliminary assumption that balancing attention between the self and other members is optimally a 1:1 distribution leads to the following hypothesis:

*Hypothesis 1: Balanced distribution of attention by SMT members to other members' contributions—defined as the theorized optimum of 1:1—will positively relate to feeling the team's work as a cohesive unit*.

With heedful contributing, the actions of individual SMT members provide the substrate for individuals to begin collectively feeling their work as a cohesive whole. To the extent that individuals develop a sense of such cohesion, they can begin to shape their contributions to that they are responsive, helping to develop a tighter, more closely interconnected web of behaviors. This, in turn, reinforces the feeling that the system of the team's work is a cohesive, rather than fragmented, whole. As more team members contribute more relevant and timely responses, the stronger their felt sense of the cohesiveness of their team's collective performance.

*Hypothesis 2: Communication responsiveness by SMT members will positively relate to feeling the team's work as a cohesive unit*.

Finally, we posit that the quality of team performance should be positively predicted by the degree to which contributing, subordinating, and representing co-occur. Moreover, the greater the sharedness of all three facets of heedful interrelating across the SMT, the better the team performance. Representing stems from contributing and subordinating, and is subsequently reinforced by them. However, if these qualities are not evident across multiple team members, then it will be difficult for team members to actually integrate their actions. As is the case with other understandings and representations of the team's work (e.g., Endsley, 1995; Lim and Klein, 2006; Rico et al., 2008), sharedness of the degree of heed across team members should facilitate better coordination, and thus better collective performance.

*Hypothesis 3: The more that members of an SMT collectively demonstrate (a) a balanced distribution of attention across their own and others' contributions, (b) responsive communications with each other, and (c) feeling the system of the team's work as a cohesive unit, the better the team performance*.

With the goal of examining the underlying mechanisms of heedful interrelating, we pursued an experimental design that removed some of the contextual “noise” of an in-depth case study, and some of the inherent difficulties in rigorously measuring individuals' micro-level attentional moves in a real-world organizational context. Based on Edmondson and McManus' (2007) framework of theory-method fit, our experimental design is an example of intermediate theory research, which involves a “cycling between inductive theory creation processes and deductive theory-testing strategies to produce and develop useful theory” (p. 1166). We enhance Weick and Roberts (1993) nascent, compelling theory with more mature ideas about attention and relationships. In our experiment, we collected rich, qualitative video data of teams coordinating in real-time, but then subjected those qualitative data to quantitative testing to test out relationships among variables. This is in keeping with the sprit of intermediate theory research which “describes a zone in which enough is known to suggest formal hypotheses, but not enough is known to do so with numbers alone or at a safe distance from the phenomenon” (Edmondson and McManus, 2007, p. 1166).

## Methods

### Participants

Two hundred and four undergraduate students at a large Midwestern university were each randomly assigned to 50 triads. Seventy-four percent of the participants self-identified as White, 11.3% identified as Asian; 7.4% as Other/non-reported; 3.3% as African-American/Black; 2.7% as Hispanic/Latino; and 1.3% as American Indian/Alaska Native. Forty-eight percent of participants were female, and only 9 teams were all male (see Table 1 for gender makeup of teams in each experimental condition). The majority of students participated for introductory psychology course credit, and a minority responded to fliers around campus advertising participation in a “Marketing Study” for $10 compensation.

**Table 1:** **Team gender composition by experimental condition**.

**Experimental condition**	**Number of all male teams**	**Number of 1/3 female teams**	**Number of 2/3 female teams**	**Number of all female teams**
Self-focused	1	5	8	0
Other-focused	3	3	4	2
Self-and-other focused	1	2	6	3
Time-focused	4	4	3	1
Totals	9	14	21	6

### Design

The experiment involved a one-by-four between-subjects design in which we attempted to manipulate attentional distribution. This was based in our theorizing that, for *ad hoc* SMTs starting to coordinate, the actions they contribute are the starting point for representing and subordinating. Individuals signing up for the study were assigned to *ad hoc* teams, based on their availability. Each team was then randomly assigned to a self-focused condition (14 teams), an other-focused condition (12 teams), a self-and-other-focused condition (12 teams), or a control condition (12 teams) in which they were told to focus on the time taken to complete the task, rather than on the self or other.

### Procedures

Upon arriving at the lab, participants were seated together at a small table, provided written consent to participate in the study, and completed a questionnaire with demographic and trait measures of various control variables. They were told a cover story maintaining that this study was part of a larger project between the Psychology and Marketing Departments about developing new commercial jingles, or short songs to sell various products provided on a list. Participants in each condition were provided with written instructions to engage in the relevant kind of attentional focus before engaging in two practice rounds of 5 min each (see Appendix A). Participants were also instructed that at the end of the task they were expected to provide evaluations of themselves, others, their joint contributions or their use of time in the task. A relevant evaluation questionnaire was distributed with the instructions.

At the start of each trial, the experimenter played the tune of a common nursery rhyme, whose familiar and simple musical structure would aid song composition. Participants were told to contact the experimenter with any questions via walkie-talkie as the experimenter left the room prior to the trial beginning to ostensibly enter some data for another study. This allowed the team to assume they were unobserved, but the experimenter observed them on a monitor in a separate room through a hidden camera and microphones. The experimenter's absence and the hiding of recording devices limited the induction of self-focus (Duval and Wicklund, 1972). After 5 min the experimenter returned to the room, halted the trial and stopped the music.

These trial rounds allowed attention to shift from initial difficulties with the task structure to the content of the contributions being made. After the two 5-min practice trials, participants re-read the task instructions in order to reinforce the attentional focus. For the third trial, participants were told to proceed in the same manner as the previous practice trials, except they would have as much time as they needed to create an eight-line song (except for the time-focused condition, in which they were also told to work as quickly as possible). The group was to “page” the experimenter with the walkie-talkie to indicate completion of the third trial. When the experimenter returned, the group completed their evaluations, and a questionnaire about their experience, which included a measure of “group feeling.” Participants were then debriefed and requests for consent for both analysis and/or display of the audio/video record of their interactions in research reports were administered.

Since contributing, subordinating, and representing are behavioral qualities, we examined the verbal and non-verbal behavior of study participants. First, research assistants transcribed the final song-composition trial of each session. It was assumed that by this trial, team members would no longer be overwhelmed with adjusting to a novel task and, having learned how to coordinate with each other in the prior trials, the mechanisms that facilitated successful coordination would be more apparent. After transcribing the entire third trial, we identified the speaking turns in the conversation where the lines that ended up in the final song were first introduced. A “speaking turn” or “turn at talk” is a concept from the field of conversation analysis, and refers to the time one speaker takes to contribute to the ongoing flow of a conversation, whether it consists of one word, phrase, sentence, or some combination of these (Drew, 2008).

Second, given the richness of the audio-video data, we had to determine meaningful limits to the focus of our analysis. Whether a team had 7 or 10 speaking “turns” in their overall conversation in which lines were introduced, we focused on the third speaking turn in which a new line was being introduced. We surmised that this point in the team's conversation should have limited influence from the prior trial or from the effects of rushing to complete the second half of the task (Gersick, 1988). With that specific speaking turn as our focal point, we looked at the 6 speaking turns prior to that focal speaking turn, and at the 6 speaking turns after that focal speaking turn. In all, each transcript contained 13 speaking turns (6 before, 1 speaking turn with the song line being contributed, and 6 speaking turns after). These final extracts, comprised of six speaking turns before and after the third contribution offered in the conversation, ranged from 24 to 186 s of speaking time, with an average speaking time of 63 s (*SD* = 33.42 s).

Third, the actions performed by participants within this extract were added to the speech transcription. Overall, this sampling procedure was based on the “thin-slicing” paradigm, which derives reliable judgments of an entire interaction from analysis of a small extract within the larger interaction (Ambady and Rosenthal, 1992; Ambady et al., 2006), and is a standard procedure in studies of coordination quality (Bernieri et al., 1988, 1996). We reviewed all the videos and transcripts to ensure clarity in the transcription of speech, actions, and physical orientation in the extract. We also segmented the transcript content into discrete portions of analyzable actions that could be coded in terms of the attentional focus they demonstrated. For example, “first you roast it then you grind it and then you pour it in like water” is a segment of transcribed speech, and “fiddles with pen throughout utterance” is a segment of transcribed action (group 101409a).

### Measures

#### Attentional distribution

**Figure 2:**
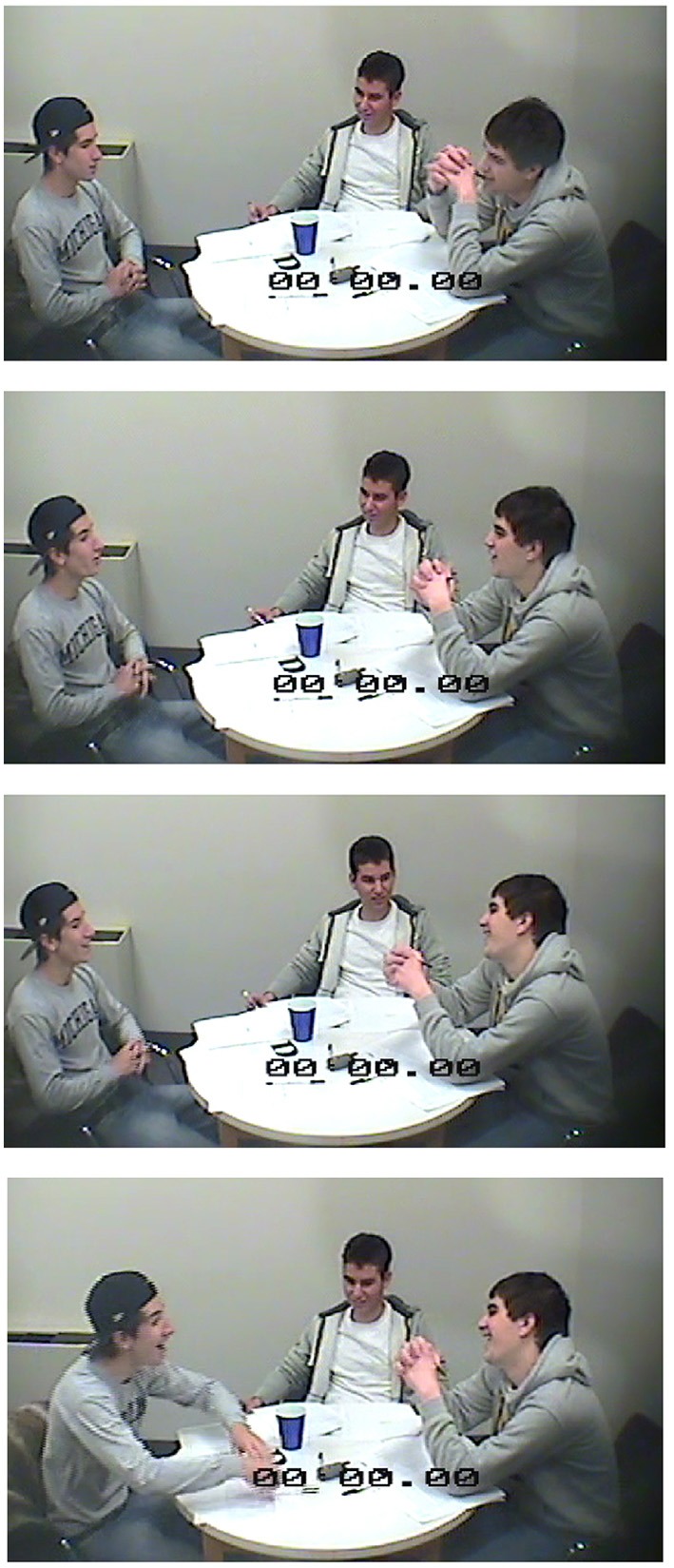
**Four-second sequence of screen grabs of self-managing team demonstrating more other- than self-focused attentional distribution**.

**Figure 3:**
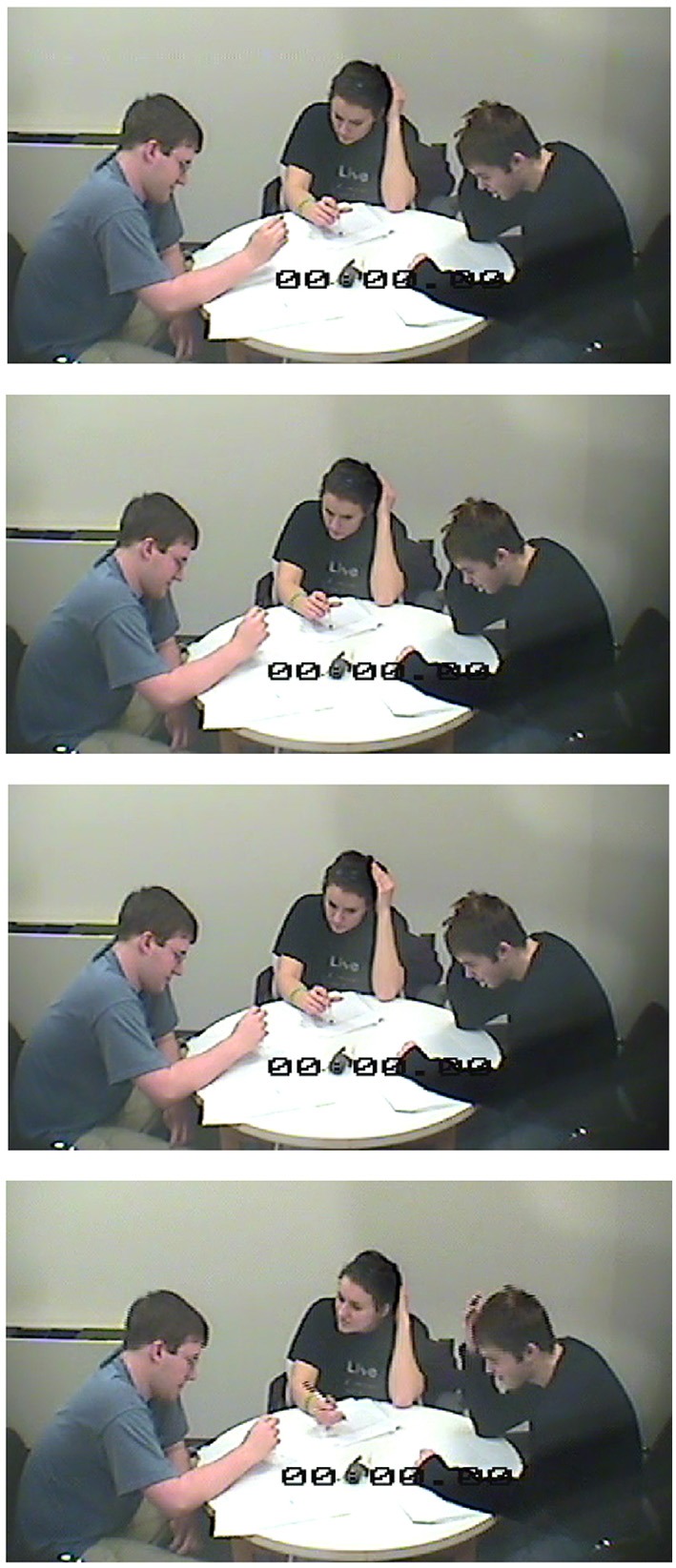
**Four-second sequence of screen grabs of self-managing team demonstrating more self- than other-focused attentional distribution**.

Attention was operationalized in terms of how it was displayed in participants' speech, gesture and bodily orientation. A coding scheme for participants' talk and action was inductively developed through a review of pilot experimental sessions, and then updated as new forms of behavior became apparent in later sessions (see Appendix B). In keeping with the advice of group process specialists who emphasize task-focused development of observational categories based on the research question at hand (Tschan et al., 2011), ecologically-valid codes were inductively developed to represent the situated behaviors associated with the task of song-composition in the lab. Each behavior was categorized in terms of how they either limited attention to the self or to others. For example, attention to the self was demonstrated in looking down at one's jingle record sheet, which limited a participant's visual attention to the words she had selected to record as part of the song, and did not allow for the receipt of visual information about the other participants. Such behaviors are less apparent in the 4-s sequence shown in Figure 2 than they are in the 4-s sequence shown in Figure 3. Similarly, attention to the other was demonstrated in nodding one's head immediately after someone else made a contribution, making public one's validation of others' efforts. Such behaviors are more apparent in the images in Figure 2, than they are in those in Figure 3. While a few behaviors were also observed that reflected a simultaneous orientation to both self and other, e.g., Supplementing another's contribution by completing their line, and spontaneously singing out the co-created contributions as a group, the total numbers of such behaviors were too small to be considered in our analyses.

Two raters uninvolved in data collection and unaware of the study's hypotheses coded the segments of speech and action in each extract. After initial clarification of the coding scheme, raters practiced coding 21 transcript excerpts of experimental sessions not included in the current study (either due to having only 2 participants, or consent to analyze, but not display audio-video data). Raters met with the first author to assess and clarify disagreements after every 5 transcript excerpts they coded. This amounted to approximately 20 h of training (practice and discussion) over the course of 6 weeks. After demonstrating moderate to strong agreement across all 21 excerpts, both raters were each provided with 30 transcript excerpts of experimental sessions with teams of 3, coding 10 of the same transcripts in order to assess reliability. To manage the amount of information that would be assessed, we aggregated subcodes for categories of self- and other-focused behaviors (15 and 17 subcodes, respectively) into two variables: self- and other-focused behaviors. Dividing self-focused by other-focused behaviors produced an individual-level ratio of self-to-other focused behaviors, and averaging this variable among group members produced a group-level self-to-other behavioral focus ratio. Higher levels of this ratio indicated more self- than other-focused behaviors as a percentage of total behaviors.

To determine if there was agreement between the two coders' judgments of the percentages of participants' behaviors that were self-focused or other-focused, we computed Krippendorff's alpha in SAS 9.4 using Hayes and Krippendorff's (2007) KALPHA macro. This measure is conservative, and accounts for chance agreements among coders. To compute alpha, individual- and group-level data files were constructed with one row per observational unit. For each variable analyzed (e.g., self-focused behavior), one column was created containing the rating for each rater (e.g., variables containing the self-focused behavior ratings of coder 1 were placed into one variable, ratings for coder were placed into a second variable). Variables were categorized as ratio variables, and all analyses were based on 5000 bootstrap samples.

The variables met standards for acceptable interrater reliability. A statistic of 0.67 is considered sufficient, and 0.8 is considered desirable (Krippendorff, 2004). There was acceptable agreement between the two raters when it came to categorizing the percentage of total behaviors that were self-focused (α = 0.78) and other-focused (α = 0.80). The individual-level ratio between self-focused and other-focused behaviors (α = 0.80) also showed acceptable reliability. Averaging this ratio for members of each group produced a group-level self-other ratio (α = 0.90) that surpassed cutoffs for acceptable reliability.

#### Responsiveness

Measures of responsiveness were derived from the transcript of the group's entire conversation in the third song-composition trial. Jeffersonian transcription conventions (Schegloff et al., 1977) were used to indicate the overlap of speech between group members (indicated with “[” in the transcript) and the latching of one participant's speech onto another's (indicated with “ = ” in the transcript). Both the overlap and latching of speech indicated the quick provision of responses to prior utterances. These measures were standardized by dividing the counts of overlaps and latchings by the number of seconds in each transcript. These standardized measures were highly positively correlated (*r* = 0.82, *p* < 0.01), so they were summed to create a single measure.

#### Feeling of wholeness of teamwork

Since representing involves feeling inhered in action, we measured it with a self-report of feeling the wholeness of the team in action. While extant measures of group cohesion implicate feeling part of a group (Seashore, 1954; Carron and Ball, 1977; Carron et al., 1985), they also contain items that capture interpersonal attraction, and a desire to remain with the group, which are distinct from tacit knowledge about the ongoing coordinating of the group. Without extant measures of feeling the system of the team's work, an eight-item measure of “team feeling” was developed, on a 5-point, Likert-type scale with anchors of *strongly disagree* to *strongly agree* (see Appendix C). Sample scale items include, “I felt ‘one’ with the group.” The internal reliability of this scale (α = 0.86) suggested it was appropriate to create a mean-level measure at the individual level (*M* = 3.93, *SD* = 0.66). Rwg values averaged 0.9, which further suggested it was appropriate to aggregate these scores to the team level, which was then used in further analyses.

#### Quality of team performance

Another pair of raters assessed the quality of the songs using a measure of “attitude toward the ad” (Biehal et al., 1992). This brief five-item measure used a 7-point Likert-type scale to acquire ratings of the “interesting” and “informative” nature of each jingle. A single new item was included to assess judgments of the coherence of the song (see Appendix C). On rating the 50 clips from the sample in the current study, the internal reliabilities of the scale for raters 1 and 2 were both strong (α = 0.87, and α = 0.93, respectively). The ICC(2) between both raters was 0.90 (*p* < 0.01), indicating a high degree of reliability across their mean responses to all six items. These raters' scores were averaged to create an overall measure of song quality.

#### Control variables

The ratio of females to males in the team, as well as a measure of interdependent self-construal (Singelis, 1994), e.g., “It's important for me to maintain harmony within my group,” were included as covariates in analyses.

Samples of the video data, the action transcripts, the quantification of the audio/video observational data coding, and the questionnaire data are available from the authors upon request.

## Results

The means, standard deviations, and correlations between the research variables at the team level are presented in Table 2, while the results of our hypothesis testing are presented in Table 3. Given the modest sample size, we report bias-corrected accelerated 95% confidence intervals based on a bootstrap sample of 5000 replications. The zero-order correlations indicated no statistically significant relationships between our measure of group-level mean attentional distribution and measures of responsiveness, team feeling, and song coherence.

**Table 2:** **Means, standard deviations, and correlations**.

	***M***	***SD***	**1**	**2**	**3**	**4**	**5**	**6**	**7**	**8**
Gender ratio	0.49	0.31								
Mean Interdependent Self-construal	3.53	0.28	0.28							
Mean S-O Attn Distr.	1.01	0.47	0.01	0.05						
Min. S-O Attn Distr.	0.57	0.31	−0.02	−0.04	0.72^**^					
Max. S-O Attn. Distr.	1.6	0.9	0.08	0.11	0.89^**^	0.39^**^				
C.o.V. of S-O Attn. Distr.	0.57	0.35	−0.13	0.06	−0.02	−0.36^*^	0.21			
Responsiveness	0.09	0.07	0.1	0.32^*^	−0.1	−0.22	0.01	−0.03		
Team feeling	3.91	0.48	0.17	0.33^*^	0.01	−0.16	0.12	0.3^*^	0.47^**^	
Judged song quality	3.99	0.93	0.13	0.04	−0.02	0.08	−0.06	−0.17	0.3^*^	0.17

Bootstrap correlations and two-tailed significance tests based on bias-corrected accelerated confidence intervals (5000 replications)

* *95% confidence interval does not include zero*.

** *99% confidence interval does not include zero*.

*For group-level correlations, N = 50*.

**Table 3:** **Summary of hierarchical regression analysis for variables predicting group-level team feeling (***n*** = 50)**.

**Variable**	**1a**	**1b**	**1c**	**1d**	**2**	**3**	**4**
Mean interdependent self-construal	0.34^*^ [0.07, 0.67]				0.31^*^ [0.01, 0.67]	0.28 [0, 0.62]	0.15 [−0.11, 0.45]
Gender ratio		0.18 [−0.08, 0.44]			0.09 [−0.21, 0.30]	0.13 [−0.16, 0.41]	0.13 [−0.12, 0.39]
C.V. of Self-other Attentional Distribution			0.29^*^ [0.05, 0.56]			0.28^*^ [0.03, 0.57]	0.31^*^ [0.09, 0.54]
Responsiveness				0.47^**^ [0.26, 0.85]			0.42^**^ [0.22, 0.78]
*R^2^*	0.11	0.03	0.08	0.22	0.12	0.20	0.36
*Adjusted R^2^*	0.09	0.01	0.06	0.20	0.08	0.15	0.30
*ΔR^2^*					0.01	0.08	0.16
*F*	6.05^*^	1.57	4.37^*^	13.25^***^	3.19^*^	3.79^*^	6.20^**^
*S.E. of Estimate*	0.95	0.99	0.97	0.89	0.96	0.92	0.84

*Standardized bootstrap coefficients and two-tailed significance tests based on bias-corrected accelerated confidence intervals (5000 replications)*.

*Confidence intervals (lower, then upper) included in brackets*.

* *p < 0.05*,

** *p < 0.01*,

*** *p < 0.001; N = 50*.

The manipulation did not produce differences in distribution of attention to self and others. One-way ANOVAs revealed no significant differences across experimental conditions in terms of how participants reported focusing on their own contributions [*F*_(3, 45)_ = 0.19, *p* > 0.1], or focusing on others' contributions [*F*_(3, 45)_ = 1.12, *p* > 0.1], or in the group-level mean attentional distribution and measures of responsiveness, team feeling, and song quality. The non-significant findings may reflect either inefficacy of the manipulation or low power. A priori analysis in GPOWER 3.1 showed that a sample of 76 teams would be needed to achieve power of 0.8 with an alpha of 0.05 and a large effect size (*f*^2^ = 0.4. However, our sample was restricted to 50 triads after dropping 5 teams that had poor video-quality or where participants declined consent to include their video records in our analysis. A *post-hoc* power analysis, assuming a large effect size of 0.4, found that a sample of 48 teams (rounded down to enable calculation), and an alpha of 0.05, would yield an estimated power of 0.59. These results suggest that participants across all conditions similarly focused on their own and on others' contributions, and coordinated similarly, despite the different instructions presented to participants in each condition. Without evidence for significant variation in self-other attentional distribution across conditions, the data from across all conditions were collapsed for analysis.

Even without differing experimental conditions to contrast, we nonetheless were able to assess how teams emergently distributed attention to self and others, and test our hypothesized relationships in a cross-sectional, rather than causal manner. Our theorizing of the impacts of attention distribution rested on assumptions of aggregating individual-level data based on their means, which “is appropriate when team members can compensate for one another with respect to task-focused contributions” (LePine, 2005, p. 33). However, the mean of teams' self-other attentional distribution displayed non-significant relationships with team feeling (β = 0.01, *p* > 0.05; 95% CI = [−0.26,0.25]), and with judged song quality (β = 0, *p* > 0.05; 95% CI = [−0.42,0.29]), thus disconfirming Hypotheses 1 and 3a, respectively. We therefore tested alternative aggregation models, which could be appropriate depending on the relationship between team functioning and performance. However, neither conjunctive (where the weakest member defines team performance) nor disjunctive (where strongest member defines team performance) aggregation models of team-level self-other attentional distribution displayed any of the hypothesized relationships with any statistical significance.

In an exploratory fashion, we computed a coefficient of variation (Harrison and Klein, 2007) for this variable, measuring the degree of group heterogeneity in individuals' self-other attentional distribution. In an alternative test of Hypothesis 1, the coefficient of variation of self-other attentional distribution was positively and significantly related to team feeling (Table 3, Model 1c; β = 0.29, *p* < 0.05; 95% CI = [0.05, 0.56]). An examination of the frequencies showed that 58 individuals across 38 teams exhibited a self-other attentional distribution greater than 1, demonstrating an aggregate propensity toward self-focused behaviors.

Hypothesis 2, which predicted that group-level responsiveness would be positively associated with team feeling was confirmed (Table 3, Model 1d: β = 0.47, *p* < 0.001; 95% CI = [0.26,0.85]). Hypothesis 3a remained disconfirmed, as the coefficient of variation of self-other attentional distribution was not significantly related to judged song quality (β = −0.16, *p* > 0.05; 95% CI = [−0.47, 0.14]). Hypothesis 3b was confirmed since responsiveness positively predicted judged song quality, [β = 0.30, *p* < 0.01; *R*^2^ = 0.09; 95% CI = [0.07, 0.59], *F*_(1, 48)_ = 4.84, *p* < 0.05].

**Figure 4:**
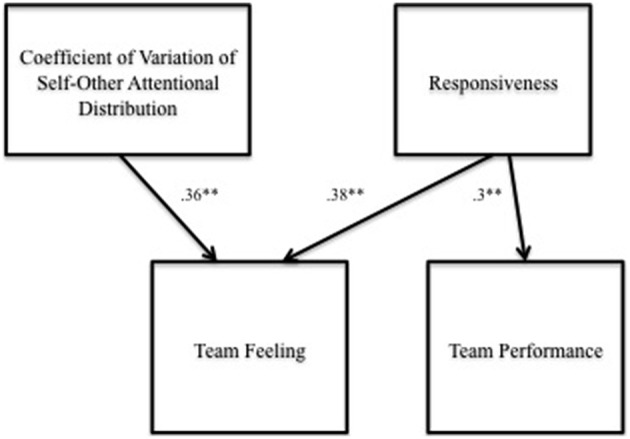
**Path model of attentive action in SMT coordination and performance**. Statistics are standardized coefficients based on bootstrap samples (5000 replications). ^**^p < 0.01.

Hypothesis 3c, which predicted that team feeling would be positively associated with judged song quality, was not confirmed (β = 0.17, *p* > 0.05; 95% CI = [−0.08, 0.41]). In a model including all the variables, the coefficient of variation of self-other attentional distribution, and responsiveness were simultaneously positively and significantly associated with team feeling over and above the control variable of interdependent self-construal (see Figure 3, Table 3, Model 4). The final path model summarizing the results of our hypothesis testing is illustrated in Figure 4.

Competing *post-hoc* hypotheses of how representing could emerge in the third trial were also tested. For example, it is possible that representing as measured by team feeling first emerged in the earlier song-composition trials, influencing what was being observed in the third trial. We tested this “learning” hypothesis but found no significant difference in the team performance (as measured by number of lines produced) in the first [*F*_(3, 43)_ = 1.42, *p* > 0.05] and second trials [*F*_(3, 44)_ = 2.29, *p* > 0.05] across conditions, nor did we find that this performance explained any variance in self-reported team feeling in the third trial (first trial lines: β = 0.22, *p* > 0.05; 95% CI = [−0.03, 0.48]; second trial lines: β = 0.17, *p* > 0.05; 95% CI = [−0.14, 0.45]). Alternatively, representing could emerge by participants managing to stay on-task, and attending to people on the team *per se*, rather than distributing attention across team members in any particular way. However, alternative models of predicting team feeling by the percentage of total behavior that was focused on self and on other (β = −0.05, *p* > 0.05; 95% CI = [−0.26,0.17]), or by the percentage of total behavior focused away from the self's and other's contributions (β = −0.11, *p* > 0.05; 95% CI = [−0.40, 0.17]), or by the ratio of non-task-focused behaviors to the total of self and other-focused behaviors (β = −0.11, *p* > 0.05; 95% CI = [−0.39, 0.16]), all demonstrated non-significant relationships.

## Discussion

In this lab study, we theorized and tested operationalizations of heedful interrelating that captured how individual members of self-managing teams (SMTs) simultaneously engaged the parts, their relationships, and the whole collective performance as they coordinated. In this way, our findings present a deeper understanding of how SMT members emergently develop their coordinating so that it leads to effective team performance. Specifically, we found that contributing and subordinating appear to predict representing as SMTs coordinate. We found that contributing as attentional distribution across self and others in the team predicted representing—feeling the system of the team's work as a whole unit—when there was within-team heterogeneity of that attentional distribution. More specifically, when one or two team members were more self- than other-focused, team members reported greater feeling of the system of the team's work as a whole unit. We also found that subordinating, measured as responsive communications within a team, independently and positively predicted the feeling of the system of the team's work as a whole unit, and the team's performance, measured as judged song quality. Finally, we found that both the variation of attentional distribution and the responsiveness of communicating were simultaneously predictive of team feeling. Our findings have significance for what we understand about how contributing, subordinating, and representing are manifest in the coordination of work teams.

First, the findings about attentional distribution within SMTs were particularly surprising. Although we found that individuals tended to have a near-equal distribution of attention across their own and others' actions (Table 2), having team members configure varying ratios of attentional distribution (Klein and Kozlowski, 2000) predicted representing. This may be reflective of the tradeoff in attentional vividness (developing a richer environmental picture through distributing attention) and attentional stability (focusing on one object at a time) noted in the literature on organizational mindfulness (Weick and Sutcliffe, 2006). These authors suggest that organizations may “distribute responsibilities for ascertaining specific objects among people in different roles or positions who remain in close contact with one another” (p. 519). The more self-focused attentional distribution of one or two individuals in 76% of the teams in our sample demonstrates the emergent, self-organizing quality of this distribution of responsibilities. The SMTs in our sample seemed to develop their own implicitly shared leadership, where individuals take turns at providing their own inputs for the sake of shaping the team's work (Carson et al., 2007). Without functional or formal power differences, SMT members, may implicitly “follow” the individual who embodies the most contributions to the group task (cf. Bunderson, 2003).

Considering our difficulty in experimentally manipulating attentional distribution also yields theoretical insights. As pointed out by an anonymous reviewer, the mix of genders across our teams may explain this (41 out of 50 teams had a female member). The women in our sample may have been more apt to attend to others while attending to themselves, negating the differential effect of instructions to focus only on the self, or only on the time taken to complete the task. Based in their greater social sensitivity in comparison to men, the more women in a group, the greater the collective intelligence of that group, or “the general ability of the group to perform a wide variety of tasks” (Woolley et al., 2010, p. 687). Similarly, research on newly-forming relationships demonstrates that conflict resolution is based in females' “softer” response to male negative affect, followed by male acceptance of female influence (Gottman et al., 1998). Future experimental studies may find it worthwhile to separate out teams by gender, to better assess the predicted relationships among variables.

All together, our findings contribute to emerging organizational literature on attention quality (e.g., Weick and Sutcliffe, 2006; Ocasio, 2011; Dane, 2013; Good et al., 2016). In our study, attention at the individual-level was distributed near-equally across self and other team members, team-level variation in this distribution shaped the felt sense of the team working as a whole, and this distributing may be influenced by gender. Moreover, although our codes for attentional behaviors were induced from the observations unique to our lab setting, the fact that they resemble extant operationalizations of heedful interrelating suggests some degree of validity. For example, Kolbe et al. (2009) induced codes from their observations of anesthesia teams at work, such as “watching actions of other team members” and “giving feedback in a positive manner,” that parallel our codes (Appendix B). However, we advance prior research by specifying how these behavioral qualities relate to specific facets of heedful interrelating. Researchers pursuing this line of qualitative inquiry into heedful interrelating should find this fine-grained approach to be revealing about the kinds of behaviors that facilitate emergent coordination, e.g., we found a low frequency of “rejecting others' contributions” in our sample, suggesting that attending to others in constructive, positive ways may be more beneficial for coordinating than criticism.

Our findings also demonstrate that among the three facets of heedful interrelating, only our measure of subordinating in terms of speech responsiveness predicted team performance, while our measures of contributing and representing did not. This suggests that our measures of the three facets are distinct from each other, and that SMT members should pay particular attention not only to their provision of mutually supportive behaviors, but also to the pace with which they mutually respond (cf. Marks et al., 2001; Burke et al., 2006). It is also possible that the pace of responsiveness reflects the influence of another unobserved causal factor, such as a shared team mental model (e.g., Lim and Klein, 2006), which was not measured in our study.

We still maintain that all three factors co-occur, since the attentional behaviors that comprise contributing precede subordinating, and the felt cohesiveness of the team's work emerges from subordinating, or the way that contributions are treated. However, all together, our results suggest that the integration of diverse contributions during coordinating seems to be especially important for the quality of the jointly-developed team product. The subtlety of our measure of responsiveness in terms of capturing the speed, rather than the content of speech, also indicates that subordinating can possibly occur quickly and non-consciously amongst group members. The concept of responsiveness in longer-term relationships suggests a certain degree of deliberation in shaping the quality of communication (e.g., Reis and Clark, 2013). Yet, similar to the swift trust that can quickly emerge in temporary team contexts (Meyerson et al., 1995), members of SMTs may need to automatically “grease the wheels” of coordinating with quick responses to each other in order to have effective performance later on.

Our findings also point toward another way in which time matters for heedful interrelating in SMTs. Although other perspectives on teamwork and coordination consider the timeliness of responses (e.g., Waller, 1999; Gittell, 2003, 2008), there is still a paucity of team-based studies that account for time (Mohammed and Nadkarni, 2011). Our research connects heedful interrelating—and specifically the facet of subordinating—to other perspectives that suggest that time matters for team functioning. For example, using the midpoint of a task as a time for transition (Gersick, 1988) helps to shape the pace at which teams execute and evaluate their taskwork, with greater familiarity among team members helping this pacing (Okhuysen and Waller, 2002). It is possible that the effects found in our study were shaped by examining how the team coordinated around the middle of their song-composition task, but it is noteworthy that these effects emerged within *ad hoc*, temporary SMTs, whose members were unfamiliar with each other. Being able to compare and contrast with other studies in this way, adds concreteness to the concept of heedful interrelating and motivates future research that can compare the influence of timeliness at different task stages in SMTs. It also corroborates features of the original theorizing that pertain to the *ad hoc* and emergent nature of coordinating as an SMT, suggesting that it is how individuals engage each other through the task, rather than their degree of interpersonal familiarity, that aids coordinating (Weick and Roberts, 1993).

Where representing is concerned, our measure of team feeling captured the intertwining of taskwork (what people do) and teamwork (how people do it; Marks et al., 2001) as members described their sense of working with others (Sandelands, 1988). With this measure, we better understand how individuals experience the sense of alignment between the parts they contribute, and the interrelation of those parts as a team-level property of ongoing activity. This suggests that, in other teams charged with creative tasks without clear criteria (Okhuysen and Waller, 2002; Hargadon and Bechky, 2006), representing the work of the team should be an important form of tacit feedback that informs participants about their coordination quality. Representing would coincide with behaviors like mutual building and extension of ideas (subordinating), and help-seeking and help-giving (contributing). Capturing how people perceive the form of their experiences working with others can be difficult, since such tacit knowledge is hard to articulate and often not encouraged in organizational settings (Taylor, 2002). Yet, our measure of team feeling demonstrates that it is not only possible to have people reliably report on their feeling of the team operating as cohesive unit, or “well-oiled machine,” but that such feelings can emerge even in an *ad hoc*, temporary SMT. This builds on the distinction made in heedful interrelating theory between a “well-developed” group that has continuously worked together for some time, and the development of the group's collective heedfulness, or “mind” (Weick and Roberts, 1993). Our findings support the notion that the quality of the immediate interrelating among team members, rather than the length of time spent together, might matter more for a sense of the team coming together as a whole.

Our findings demonstrate the potential for operationalizing each of the facets of heedful interrelating, and empirically articulating their independent and joint effects on team performance. We believe it is also the first attempt to study heedful interrelating in SMTs performing a creative task, which generalizes this theory to an important new context and type of performance outcome. Our study joins the few that have captured how the quality of interactions between individuals aids a collective creative process over time (e.g., Hargadon and Bechky, 2006; Harrison and Rouse, 2015). We encourage future research that builds on our approach to further clarify the nature, impacts, and management of heedful interrelating.

### Practical implications

Concretizing the facets of heedfully interrelating reveals key insights for management practice. Most importantly, maximizing heed appears to involve a mixture of leadership and followership in directing attention and timely, responsive communication, which in turn yield a felt sense a team. Managers seeking to maximize heedful team interactions should focus on these aspects of coordination.

Our data suggest SMTs may benefit from developing or drawing on extant feedback structures to guide their contributing and subordinating. For example, the decentralized nature of SMTs may require self-imposed turn-taking in shared leadership, and having different team members contributing to the team in different ways. Since responsively communicating may be difficult to actively monitor, given its rapid and embodied nature, SMT members will need to discover ways to examine the degree of fluidity, lags, or disruption within the team. Where team processes are embodied and recorded, e.g., in surgical, sports, or military teams, internally-appointed observers or leaders can facilitate review of video-records of actual or simulated team performances (e.g., Morrison and Meliza, 1999), evaluating the team's process in terms of speed or fragmentation of the task. More continuous real-time monitoring, rather than reflecting and adapting at halfway points or through creating their own interruptions (e.g., Gersick, 1988; Gersick and Hackman, 1990; Okhuysen, 2001; Okhuysen and Waller, 2002), should better inform SMT members about the quality of their collective coordination. Like a canary in a coal mine, a lack of responsive communication may serve as a real-time bellwether of group disintegration and performance; continuous responsive communication should predict group coherence and coordination.

The attentional distribution and responsiveness that feed into team feeling may be aided through various material supports. Representing appeared to be aided in our study by SMT members drawing on available, tangible, sensory mechanisms. These mechanisms may have helped to make the responsive communications more explicit and visible for participants, as well as provided an object of shared attention within the group, which facilitates greater processing and pursuit of the goals associated with the object (Shteynberg and Galinsky, 2011). For example, participants collectively used the music being played, and their simultaneously-updated song record sheets as common resources through which they could anchor their joint focus (e.g., Metiu and Rothbard, 2012) and sense their performance as a whole. In formal work organizations, where SMTs may have representations like a common protocol to guide them in a new task, written or drawn protocols should be jointly-designed and updated. Rather than static boundary objects that are unmodifiable by team members (Carlile, 2002), collectively-designed representations may be especially important for bridging differences in understanding amongst SMT members with different backgrounds (e.g., Dougherty, 1992; Bechky, 2003). Finally, while many virtual teams in organizations schedule occasional face-to-face or video team meetings, our results suggest that intentionally engaging the entire team in embodied behavior to work through issues e.g., in simulations, may be helpful to assess the sharedness of individuals' representations.

### Limitations and future research directions

The generalizability of our results is limited in several ways. The embodied nature of our theoretical and empirical focus may limit our results to teams whose members work face-to-face. This may be important to consider, given that organizations are increasingly turning to virtual teams, whose members and actions are distributed across space and time, to handle increasing complexity (Gilson et al., 2015). Observable behaviors like intertwining speech, gaze, body orientation, and gesture may be more easily and consistently accessed by group members involved in co-located physical tasks (e.g., sports teams). Yet, there is evidence for the importance of face-to-face interaction for members of virtual teams and communities, for socializing, taskwork, and establishing authority (Crowston et al., 2007; O'Mahony and Ferraro, 2007). Thus, while our work is surely focused on understanding the coordination within “in-person” teams, we suggest that outlining the dynamics of these kinds of teams is critical for better appreciating the subtle dynamics that might be critical for the success of virtual teams whose members periodically meet “in the skin” or via videoconferencing.

Also, the failure of the experimental manipulations means that the relationships among our variables must be considered cross-sectional, not causal. We cannot rule out alternative explanations for the relationships found in our study, such as the possibility that some unobserved factor determined the emergent attentional distribution along with other variables. Finding other ways to manipulate the strength of contributing, subordinating, and representing in teams will be necessary to effectively compare and contrast variations in heed, and what outcomes they predict. In addition to contrasting teams of different genders, future designs may help to tease out the causal sequence in which heed influences team processes and outcomes by perhaps comparing teams with unlimited physical access to each other with those who have varying degrees of co-location. Introducing conditions with different levels of task or communication disruption (e.g., LePine, 2005) may also be used to shape the degree of heed and the subsequent quality of the team's coordination.

Despite these limitations, our results call for further research around heedful interrelating in teams. They suggest that further theorizing is needed about why heterogeneity in a team's self-other attentional distribution is positively associated with representing the system of the team's collective work. The empirical relationships among our operationalizations of contributing, subordinating, and representing present a first step toward demonstrating internal reliability, and thus the validity of the overall heedful interrelating construct, that can form the basis for further validity testing. We also have begun establishing predictive validity of one facet of heedful interrelating, as subordinating in the form of responsive communication predicted team performance. Future efforts can be directed toward building a nomological network of antecedents (psychological safety, Edmondson, 1999; e.g., trust; McAllister, 1995), parallel constructs (e.g., relational coordination; Gittell, 2002), and outcomes (e.g., patient health outcomes; Edmondson et al., 2001; Vogus and Sutcliffe, 2007) to further establish validity.

Other research can better account for longitudinal effects on heedful interrelating in SMTs. Although we accounted for the effect of learning in our analyses, future research can more closely examine the role of early interactions in SMT development. The resources required to then maintain the contributing, subordinating and representing observed in our study are also less-well understood, although prior research suggests that heedful interrelating may also fade away over time without supporting contextual factors (Druskat and Pescosolido, 2002). The traits individuals bring into coordinating may also be important to examine, as interdependent self-construal appeared to have a considerably positive, predictive relationship with team feeling in our study. While we focused on components of the dynamic process of coordination, rather than static traits, this suggests that there may be other relationally-oriented traits worth exploring.

To conclude, by operationalizing contributing as attending, subordinating as responsively communicating, and representing as feeling the whole of the system of the team's work, this study shows how individuals can simultaneously engage in the parts, interrelationships, and whole of coordination. Self-managing teams can demonstrate a full engagement in intelligent, heedful action that generates the careful, and purposive teamwork on which so many organizations rely.

## Author contributions

Data in this article were collected as part of the first author's dissertation, and he thanks Lloyd (Lance) Sandelands for his guidance. We are also grateful to members of the Coordination Lab at the University of Michigan (Antonio Adan, Kate Balzer, Kellen Brackins, Amanda Burnett, Caraline Craig, Nathan Fink, Sarah Francus, Shannon Herline, Maria Johnson, Cliopatra Kilonzo, Lauren Levine, and Emily Nadis) and at Case Western Reserve University (Inho Choi, Yung Mo Cho, Meredith Dykehouse, Andy Lai, Adam Lerner, Skyler Phillips, Njoke Thomas, Rebecca Vaughan, and Hongguo Wei) for their assistance with data collection and analysis.

## Funding

The first author received a Dissertation/Thesis Grant from the Department of Psychology and a Graduate Student Research Grant from the Horace H. Rackham School of Graduate Studies at the School of Michigan.

### Conflict of interest statement

The authors declare that the research was conducted in the absence of any commercial or financial relationships that could be construed as a potential conflict of interest.
