# Supplementary material for: Operationalizing Heedful Interrelating: How Attending, Responding, and Feeling Comprise Coordinating and Predict Performance in Self-Managing Teams
Source: Front Psychol. 2016 Mar 18;7:362. doi: 10.3389/fpsyg.2016.00362 (PMC4796034; doi:10.3389/fpsyg.2016.00362)
Supplement: Supplementary file 1 [file DataSheet1.docx]

**APPENDIX A**

Attentional manipulation instructions

In the *self-focused attentional* condition, instructions about the task read:

Your task in today’s study is to create a song about a common household product. Like a team of advertisers that produces jingles for commercials, as a group you all must together come up with a song for one of the products in the booklet on the table. You are to collaborate with others in the group, creating and sharing your contributions with them in order to quickly generate a complete song. Your goal as a group is to create the best song possible about the product. Each person in the group will be held responsible for an equal portion of the work. Your goal as an individual is to *make the best contributions that you can. You will evaluate your own contributions***.** The tune for the jingle will be played in the background to help with the task. Please use the forms provided to record the lines to the song, and follow the instructions on them as well.

In the *other-focused* condition, the relevant instructions were: “*help others make the best contributions that they can. You will evaluate their contributions*.”

In the *self- and other-focused* condition, the relevant instructions were: “*relate your contributions to the contributions of others in the best way possible. You will evaluate your joint contributions.*”

In the *time-focused* condition, the relevant instructions were “*keep in mind how long the group takes to complete the task. You will evaluate your use of time in this task.”*

**APPENDIX B**

Codes for analysis of attentional behaviors in action transcripts

| Attentional Focus Category and Code | Behavioral Samples |
| --- | --- |
| Verbal non-task focus | Behavior that includes discussing anything other than how to create the song, or providing words, ideas or lines that will help add to/generate the song, e.g. talking about doing other studies, or possibility of being watched. |
| Non-verbal non-task focus | Behavior that includes engaging with materials other than those provided in the experiment, e.g. playing with laptop, checking cell phone. This also includes looking up and away from people, whether towards the wall, or towards the camera |
| Attention to self |  |
| Looking | Looking down at one’s record sheet, without writing. |
| Touching/Shuffling | Moving one’s papers around, picking them up etc. |
| Requesting audience | Saying things like, “Do you want to hear mine?” |
| Suggesting | Saying “How about…?” |
| Contributing | Saying out own ideas or lines to song (with or without writing them down first, and without following another’s question or line |
| Separately contributing | Writing out own lines then sharing with group |
| Non-verbal acceptance | Writing down the line(s) one contributes to group |
| Rejecting (oneself) | *Contributing*, or *verbally answering* but then immediately saying “...but that is (would be) awkward/dumb…” or physically pulling away from group members |
| Verbal Questioning (of self) | Saying “Does that sound right?” immediately following self-contribution |
| Self-modification | Re-wording self-contributed line immediately after contribution (not following another’s contribution) |
| Quiet singing | Singing or mouthing out words to background music to self. |
| Adaptor gestures | Scratching, shifting clothes, flipping hair back, coughing, putting pen in mouth, chewing nails |
| Holding/Claiming floor | Saying “Umm,” or “Wait” before pause in speech or maintaining a gesture while paused in speech |
| Non-relevant transition | Changing topic when response to it is expected |
| Verbal self-acceptance | Saying “I like what I just said” |
|  |  |
| Attention to other |  |
| Postural physical orientation | Turning one’s upper body towards another person. |
| Head and face physical orientation | Turning only one’s head or face towards another person. |
| Looking | Looking down at others’ record sheet, or at another person |
| Touching | Reaching out and/or touching another person’s record sheet |
| Verbal questioning | Asking questions about what others are thinking or would like to say (prior to any contribution being offered |
| Verbal answering | Responding to questions asked by others |
| Non-verbal answering | Nods or shakes of head, shrugging of shoulders immediately following verbal questioning |
| Sharing of record sheet | Re-positioning one’s record sheet so another could see its contents |
| Moving | Moving chair or body physically closer to another |
| Verbal acceptance | Saying “Alright!” in response to another’s contribution |
| Non-verbal acceptance | Writing others’ contributions OR smiles, nods that: (1) immediately follow a contribution and (2) are themselves followed by speech or action that confirms/validates |
| Verbal Rejection | Saying “That’ll sell” sarcastically |
| Non-verbal rejection | Not writing other’s contributions at all; physically pulling away from others in responding to their contributions |
| Visibly gesturing | Performing hand gestures that indicate rhythm of music, pointing at someone, counting with fingers |
| Audibly singing | Singing out words to background music to others [must include eye contact with another] |
| Seeking validation | Asking “Right?” or “Don’t you think?” after making a contribution |
| Giving rationale | Providing rationale behind one’s contribution |

**APPENDIX C**

1. Scale items for judged song quality

Please use the scale below to indicate what you think about the jingle along the following dimensions:

1. Bad….Good
2. Dislike…Like
3. Boring…Interesting
4. Uncreative…Creative
5. Uninformative…Informative
6. Incoherent jumble of lines…Well-integrated lines

2. Scale items for team feeling

1. I felt that I was in harmony with others
2. I felt as if I was working by myself but others happened to be present.
3. I felt “one” with the group.
4. I felt part of something greater than myself.
5. I did not feel like this was truly a group effort (reverse-scored).
6. I felt like I was “in a groove” with others.
7. I did not feel as if I was “one” with others (reverse-scored).
8. I felt like a true member of a team
